# Microvessel density and heparanase over-expression in clear cell renal cell cancer: correlations and prognostic significances

**DOI:** 10.1186/1477-7819-9-158

**Published:** 2011-12-02

**Authors:** Juchao Ren, Hainan Liu, Lei Yan, Sujian Tian, Dawei Li, Zhonghua Xu

**Affiliations:** 1 Department of Urology, Qilu Hospital, Shandong University, 107# Wenhua Xi Road, Jinan, 250012 P.R. China; 2 Key Laboratory of Cardiovascular Remodeling and Function Research, Chinese Ministry of Education and Chinese Ministry of Public Health, 107# Wenhua Xi Road, Jinan, 250012 P.R. China

**Keywords:** clear cell renal cell carcinoma, microvessel density, heparanase, prognosis

## Abstract

**Background:**

Tumor angiogenesis is important in the progression of malignancies, and heparanase plays an important role in sustaining the pathology of clear cell renal cell cancer (ccRCC). The study was carried out to investigate the correlations between microvessel density (MVD) and heparanase expression containing prognostic significances in the patients with ccRCC.

**Methods:**

Specimens from 128 patients with ccRCC were investigated by immunohistochemistry for MVD. RT-PCR and immunohistochemistry were used to detect heparanase expression. Correlations between MVD, heparanase expression, and various clinico-pathological factors were studied. The prognostic significances of MVD and heparanase expression were also analysed.

**Results:**

We discovered a statistically significant prevalence of higher MVD in ccRCC compared with adjacent normal renal tissues. MVD was positively correlated with TNM stage and distant metastasis in ccRCC patients, and was also correlated with the expression level of heparanase.

Heparanase is over-expressed and correlated with TNM stage, histologic grade, distant metastasis and lymphatic metastasis in ccRCC. High MVD and heparanase over-expression inversely correlate with the survival of ccRCC patients.

**Conclusions:**

Heparanase contributes to angiogenesis of ccRCC and over-expression of heparanase is an independent predictors of prognosis for ccRCC. MVD is correlated with tumor development and metastasis in ccRCC.

## Background

Renal cell carcinoma (RCC) is the most common malignant tumor of adult kidney [1]. Approximately 30% of the patients will develop to metastatic disease after curative surgery [2]. Clear cell renal cell carcinoma(ccRCC) is the most pathological types of RCC which has various clinical-pathological characteristics and prognostic factors. Angiogenesis is one of the major factors in the progression of malignancies, and tissue angiogenesis can be quantified by counting microvasculars in a certain area by immunohistochemical staining (microvessel density, MVD) [3]. So far MVD has been considered as a potential prognostic marker in some tumors [4,5].

Heparanase(HPA) is an endo-β-D-glucuronidase that has the activity of cleaving heparan sulfate (HS) side chains of heparan sulfate proteoglycans (HSPGs) [6]. HSPGs are not only the major proteoglycans of the extracellular matrix (ECM) and basement membrane (BM) which play a key role in preventing tumor cells invasion and metastasis, but also expressed on cell surfaces [7]. It is well known that heparanase activity is concerned with angiogenesis, inflammation, and cancer metastasis [8]. Heparanase over-expression inversely correlates with survival of patients with gastric [9], pancreatic [10], cervical [11], colorectal [12], bladder [13] and prostate [14] cancer.

The description about the relationship between heparanase and MVD has not been so far done in previous studies. Our study examined MVD and heparanase expression in 128 patients with ccRCC and analyzed the correlations between the clinical-pathological parameters including MVD and heparanase expression. Moreover, we analyzed the prognostic significances of MVD and heparanase expression for ccRCC.

## Methods

### Patients

The tumour specimens and corresponding normal renal tissues were obtained from 128 patients with ccRCC between 2002 and 2008. All the ccRCCs were staged according to the 1997 TNM staging system [15]. Nuclear grade was on the basis of the Fuhrman criteria [16]. Clinical data of all the patients were collected from hospitalization and subsequent records. All the patients were informed of the study and consented to using their renal tissues for the investigation. Our study was also approved by the local ethics committee. All the patients were underwent radical nephrectomy, and none of them received chemotherapy or radiation therapy before surgery. The specimens were stored in liquid nitrogen for RT-PCR, and paraffin-embedded sections were prepared for immunohistochemistry analysis. We were keeping follow-up of 70 patients, 28 of them were alive at the end of the follow-up. Detailed information is listed in Table 1.

**Table 1:** MVD and Expression of HPA According To Clinical-pathological Parameters in ccRCC Tissues

Variables	*n*	%	HPA		*P*	MVD	*P*
						
			-	+			
Total	128	100	32	96		101.64 ± 23.00	
Gender							
Male	82	64.1	21	61	0.832	101.39 ± 24.54	0.133
Female	46	35.9	11	35		102.07 ± 20.04	
Age, Years (Median 55)							
≤ 55	61	47.7	15	46	0.919	102.02 ± 20.49	0.069
> 55	67	52.3	17	50		101.27 ± 25.14	
Tumor size							
≤ 5 cm	83	64.8	20	63	0.748	101.93 ± 24.53	0.058
> 5 cm	45	35.2	12	33		101.11 ± 19.92	
Tumor stage							
T_1_	50	39.1	20	30			
T_2_	41	32	8	33			
T_3_	27	21.1	3	24			
T_4_	10	7.8	1	9			
Lymphatic metastasis							
N_0_	108	84.4	31	77	0.025	101.81 ± 23.84	0.056
N_1-3_	20	15.6	1	19		100.72 ± 17.91	
Distant metastasis							
M_0_	114	89.1	32	82	0.022	99.96 ± 23.66	< 0.0001
M_1_	14	10.9	0	14		113.14 ± 12.88	
TNM stage							
I-II	76	59.4	27	49	0.001	95.96 ± 21.39	< 0.0001
III-IV	52	40.6	5	47		108.94 ± 22.94	
Histologic grade							
G_1_-G_2_	94	73.4	27	67	0.106	101.49 ± 24.02	0.238
G_3_-G_4_	34	26.6	5	29		102.04 ± 20.00	
Venous invasion							
Negative	106	82.8	26	80	0.787	101.79 ± 23.99	0.109
Positive	22	17.2	6	16		100.91 ± 17.61	

-: negative expression +: positive expression

### Immunohistochemistry

Five-micron paraffin sections were prepared for the experiment. Staining of the sections for heparanase was performed as previously described [17]. SP immunohistochemistry method and DBA staining were performed. Block nonspecific binding was performed with 5% bovine serum albumin(BSA) for 60 minutes at room temperature, without washing, and the slides were incubated with anti-heparanase antibody (sc-25825, 1:100 dilution) and anti-CD34 antibody (sc-19621, 1:100 dilution) at 4°C overnight. As a negative control, the primary antibody was replaced with phosphatebuffered saline (PBS). After washing with PBS, the slides were incubated with horseradish peroxidase-conjugated goat anti-rabbit IgG according to the manufacturer's instructions at 37°C for 45 min. Color was developed with DAB Horseradish Peroxidase Color Development Kit (P0202, Beyotime). The slides were evaluated by two independent pathologists who were blind to the status of the patients according to the intensity of cancer cells that were stained (-: negative, +: weak, ++: strong) as described [18].

### Quantification of Microvasculature Density

**Figure 1:**
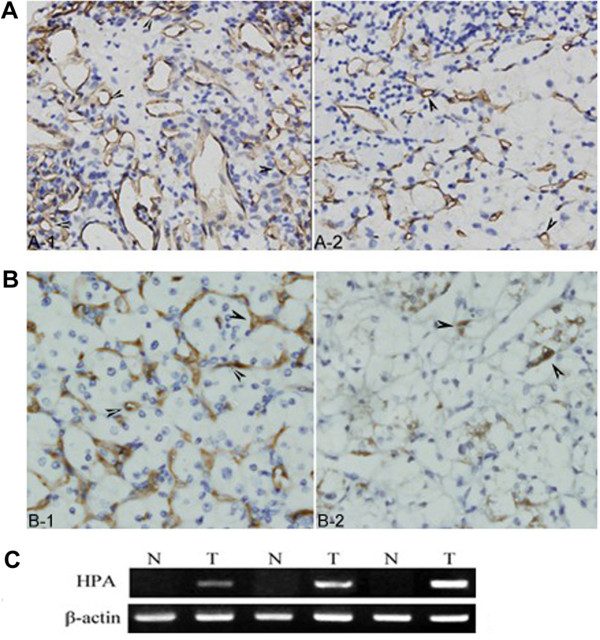
**MVD and heparanase expression in ccRCC**. (A) MVD in ccRCC tissues of immunostaining (×400). A-1: high MVD, A-2: low MVD (B) Heparanase protein expression in ccRCC tissues of immunostaining (×400). B-1: strong expression, B-2: weak expression. (C) Heparanase mRNA expression in ccRCC tissues (T) and corresponding adjacent normal renal tissues (N).

**Figure 2:**
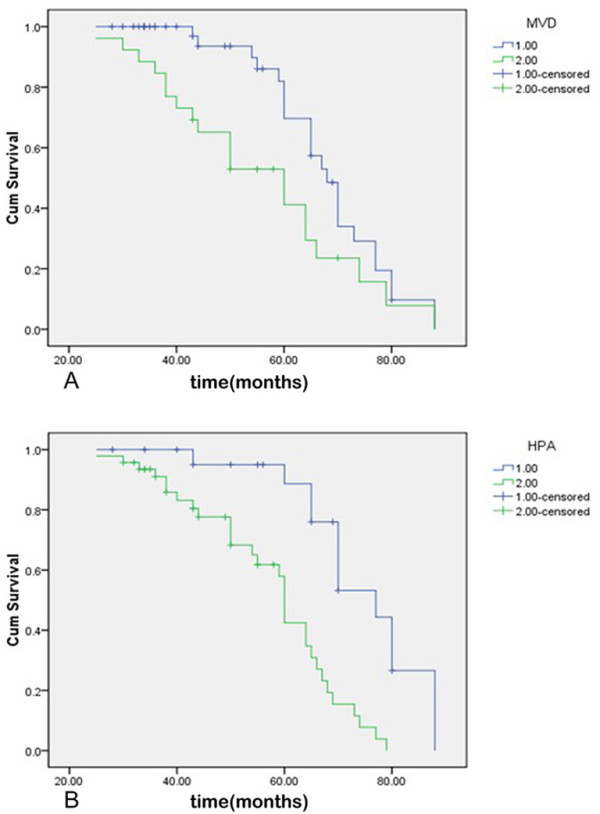
**Survival Curves of the Patients with ccRCC**. (A) on the basis of MVD counts in tumor tissues, 1: low MVD group, 2: high MVD group (B) on the basis of HPA expression in tumor tissues, 1: negative expression group, 2: positive expression group.

The primary antibody against CD34 was used to evaluate microvessel density (MVD), which was performed according to the methods previously described [19]. Endothelial cell clusters and endothelial cells which were stained brownish-yellow could be considered as a single microvessel. Undefined endothelial cell fragments and the visible vascular lumen were not counted as microvessels. Branching structures were considered as single vessel if there was not a break in continuity of the structure. Initially, the entire section was scanned for high-MVD areas by low microscopic magnification (40× and 100×) to identify the hot spots (representing the highest vascular density), and then brown-stained endothelial clusters were counted under light microscope observation 200× magnification. Results were shown as mean microvessel counts of the three hot spots under 200× magnification [20]. High MVD group and low MVD group were classified by the the median MVD counts of ccRCC patients. Figure 1A-1 showed a representative field of high MVD in ccRCC. For comparison, Figure 1A-2 depicted a representative field of ccRCC with low MVD.

### RNA isolation and semi-quantitative reverse transcriptase polymerase chain reaction (RT-PCR)

Total RNA was extracted from RCC tissues and adjacent normal renal tissues using TRIzol (Invitrogen) according to the manufacturer's instructions, and then quantified by spectrophotometry. The polymerase chain reaction (PCR) primers designed by Premier Primer 5.0 software were as following: heparanase-5'-TTCGATCCCAAGAAGGAATCAAC-3' (forward) and 5'-GTAGTGATGCCATGTAACTGAATC-3'(reverse), and β-actin-5'- GTGGGG CGCCCCAGGCACCA-3'(forward) and 5'-CTCCTTAATGTCACGCACGATTTC-3'(reverse). PCR products were separated by electrophoresis through a 1.5% agarose gel, stained by ethidium bromide, and visualized in ultraviolet light. The intensity of the bands was quantified with Scion Image software (Scion, Frederick, MD).

### Statistical Analysis

MVD differentials were compared using t-test and univariate analysis of variance. Associations between heparanase expression and clinical-pathological parameters were analyzed using Chi Square test. Univariate association of survival was evaluated using Kaplan Meier curves, and tested by Log-Rank test. Multivariate analyses were performed according to Cox proportional hazards regression mode. (SPSS version 16.0 for Mac; SPSS, Inc., Chicago, IL, USA). P < 0.05 was considered to be statistically significant.

## Results

### The correlation of MVD in ccRCC with the clinical-pathological parameters

The mean MVD counts were 101.64 ± 23.00 in ccRCC tissues, 32.52 ± 7.85 in corresponding normal renal tissues, with significant difference (*P *< 0.0001); The relationship between MVD counts and various clinical and histopathologic parameters was analyzed. The mean MVD counts were 108.94 ± 22.94 in advanced stage group (stage III-IV), 95.96 ± 21.39 in early stage group (stage I-II), with significant difference (*P *< 0.0001); 113.14 ± 12.88 in M_1 _group (with distant metastasis), 99.96 ± 23.66 in M_0 _group (without distant metastasis), with significant difference (*P *< 0.0001); No significant association was demonstrated between MVD counts and patients' age (≤55 years:102.02 ± 20.49, >55 years: 101.27 ± 25.14, *P *= 0.069), gender (m: 101.39 ± 24.54, f: 102.07 ± 20.04, *P *= 0.113), tumour sizes (≤5 cm:101.93 ± 24.53, >5 cm: 101.11 ± 19.92, *P *= 0.058), histologic grade (G_1_-G_2_: 101.49 ± 24.02, G_3_-G_4_: 102.04 ± 20.00, *P *= 0.238), lymphatic metastasis (N_0_: 101.81 ± 23.84, N_1-2_: 100.72 ± 17.91, *P *= 0.056) and venous invasion (V_0_: 101.79 ± 23.99, V_1_: 100.91 ± 17.61, *P *= 0.109) (Table 1).

### Heparanase is over-expressed and correlated with progression and invasion in ccRCC

Expression of heparanase was observed in 96 tumour samples (75%) (Figure 1B and 1C), conversely in 25 adjacent normal tissues (19.5%) (*P *< 0.0001). Heparanase expression was correlated with TNM staging (*P *= 0.001), distant metastasis (*P *= 0.022) and lymphatic metastasis (*P *= 0.025) (Table 1).

### Correlations between MVD and heparanase expression

The mean MVD counts in the group of heparanase-negative expression, weak heparanase expression and strong heparanase expression were 97.37 ± 21.13, 101.86 ± 22.53 and 103.97 ± 24.10 (*P *< 0.0001), respectively.

### MVD, heparanase expression and postoperative survival of the patients

The overall survival (OS) rate of patients with high-MVD was significantly lower than that with low-MVD (Log-Rank Test, *P *= 0.022, Figure 2A). The OS rate of patients with heparanase- positive was significantly lower than that with heparanase-negative (Log-Rank Test, *P *< 0.0001, Figure 2B). In addition, the multivariate analysis indicated that heparanase-positive expression (*P *= 0.006) and distant metastasis (*P *< 0.0001) were independent prognostic factors for ccRCC patients (Table 2).

**Table 2:** Cox regression analysis in overall survival of RCC patients

Risk factors	RR	95%CI	*P*
Primary tumor stage			
T_1, 2_	1.579	0.763-3.266	0.218
T_3, 4_			
Distant metastasis			
M_0_	5.859	2.186-15.706	< 0.0001
M_1_			
HPA staining			
Negative	3.584	1.448-8.870	0.006
Positive			
MVD			
Low	0.930	0.453-1.911	0.844
High			

RR: relative risk; 95% CI: 95% confidence interval

Rates and P values in disease-specific survival according to the status of MVD and heparanase expression were actually equal to those in overall survival of the patients.

## Discussion

As shown in previous studies, heparanase plays an important role in sustaining the pathology of various malignancies [14,17,21-24]. Invasion and metastasis of tumor cells depend on the ability of the cells invading tissue barriers which are composed of BM and ECM. HS and HSPGs, as crucial structural components, are contained in BM and ECM, and are substrates of heparanase that is capable of cleaving HS side chains [25]. Consequently, tumor cells are facilitated penetrating the BM and ECM barriers. This enzymatic activity of heparanase contributes to the development of ccRCC. Thus, heparanase may play biological roles in multiple ways other than its enzymatic activity, such as mediation of cell adhesion [26], activation of CD44 variant exon-3 [27], promoting VEGF expression via Src pathway [28], enhancing Akt signaling pathway and stimulating PI3K- and p38-dependent endothelial cell migration and invasion [29], modulation of endothelial cell permeability and integrity via syndecan family members [30,31], promotion of basic fibroblast growth factor releasing and inhibition of activated T lymphocytes [32], and so on.

Degradation of the subendothelial BM, migration and proliferation of the endothelial cells to form vascular sprouts are crucial early events during angiogenesis [33]. The activities of heparanase and some growth factors binding HS, such as aFGF (acid fibroblast growth factor), bFGF (basic fibroblast growth factor), VEGF (vascular endothelial growth factor) are directly involved in above progresses [28]. Furthermore, cell surface HS can interact with HS binding growth factors and facilitate the signal transduction [34]. Heparanase promotes migration and proliferation of the endothelial cells by above mechanism [25]. Accordingly, we evaluated the angiogenesis of ccRCC by detecting commonly used pan-endothelial marker (CD34) as previous studies [5,35].

Our study found the prevalence of elevated angiogenesis and heparanase over-expression in ccRCC, at the same time, we firstly described the close correlations between angiogenesis and heparanase expression in ccRCC. Tumor angiogenesis was closely related to the development and metastasis of ccRCC, and heparanase over-expression was associated with invasion and prognosis that was consistent with previous description [36]. Above results indicated that antiangiogenesis by inhibiting heparanase maybe more effective treatment for ccRCC.

The study showed poor survival of patients with high MVD and heparanase over-expression. Tumor size, tumor stage, nuclear grade, and metastasis status have been reported to be of prognostic significance for RCC [37,38]. Our study showed that high MVD had prognostic significance for ccRCC, which was not consistent with previous description [39].

In view of the above, heparanase may serve as a therapeutic target for ccRCC. In fact, PI-88, a representative heparanase inhibitor, has already been tested in clinical trial [40-42]. The application of heparanase inhibitors may be a promising therapeutic tool for ccRCC in particular.

Taken together, our findings demonstrate the tumor angiogenesis and the role of heparanase in ccRCC on the basis of clinical and pathological parameters, thus contributing to the basic understanding of the most common malignant tumour of adult kidney. In addition, the role of heparanase in tumor angiogenesis, the prognostic significance of heparanase over-expression for ccRCC are described.

## Conclusions

Elevated angiogenesis is observed in ccRCC but almost absent in normal renal tissues. Elevated angiogenesis contributes to development and invasion of ccRCC. Heparanase over-expression is prevalent in ccRCC, which promotes tumor angiogenesis and indicates worse prognosis. Moreover, heparanase over-expression is an independent predictor of prognosis for ccRCC. Therefore, heparanase may serve as a potential therapeutic target for ccRCC.

## Abbreviations List

MVD: microvessel density; HPA: heparanase; RCC: renal cell carcinoma; ccRCC: clear cell renal cell carcinoma; ECM: extracellular matrix; BM: basement membrane; HS: heparan sulfate; HSPGs: heparan sulfate proteoglycans; BSA: bovine serum albumin; OS: Overall survival.

## Competing interests

The authors declare that they have no competing interests.

## Authors' contributions

JCR and HNL carried out experimental procedures and drafted manuscript.. LY, SJT and DWL participated in the study design and were involved in the collection of clinical data and specimens. ZHX guaranteed the whole study. All authors read and approved the final manuscript.

## Authors' information

^1^Department of Urology, Qilu Hospital, Shandong University, Jinan, P.R. China

^2^Key Laboratory of Cardiovascular Remodeling and Function Research, Chinese Ministry of Education and Chinese Ministry of Public Health, Jinan, P.R. China
